# Clinically Relevant Topics and New Tendencies in Childhood Nutrition during the First 2 Years of Life: A Survey among Primary Care Spanish Paediatricians

**DOI:** 10.3390/nu16132146

**Published:** 2024-07-05

**Authors:** Ana Martín-Adrados, Amalio Fernández-Leal, Jorge Martínez-Pérez, Jesús Delgado-Ojeda, Alicia Santamaría-Orleans

**Affiliations:** 1 Gastroenterology and Nutrition Service, Hospital Infantil Universitario Niño Jesús, 28009 Madrid, Spainamalioafernandez@gmail.com (A.F.-L.); jorgemartinezperez@hotmail.com (J.M.-P.); 2 Medical Department, Laboratorios Ordesa S.L., 08830 Sant Boi de Llobregat, Spain

**Keywords:** infant nutrition, young children nutrition, baby-led weaning, vegetarian diet, organic food, complementary feeding

## Abstract

A multicenter cross-sectional study was conducted among 245 experienced Spanish paediatricians, who completed an online survey based on clinically relevant topics in nutrition during the first two years of life and their recommendations to parents in daily clinical practice. Most participants advise about the choking risk associated with baby-led weaning (BLW) and more than 60% consider that infants can receive an insufficient variety and quantity of nutrients with this practice. The general opinion is that there is a lack of evidence for delaying the introduction of gluten and other allergenic foods in the complementary feeding of healthy infants. Most participants agree/strongly agree that two servings of dairy products are the adequate daily amount in a diversified diet and 93.4% disagree/strongly disagree with the use of vegetal beverages under 1 year of life. There is a general agreement to avoid added salt and sugar before 12 months of life, the consideration that organic foods do not have a better nutritional profile than non-organic ones, and the limitations of vegetarian diets especially for adequate provision of micronutrients. Overall, there is an adequate knowledge of the new trends by paediatricians and younger ones seemed more in favor of them and interested in receiving more information on most topics.

## 1. Introduction

The first two years of life represent a critical window for establishing an optimal nutritional intake that profoundly influences children’s growth, development, and long-term health outcomes. Qualitative and quantity nutrient intakes during this period play a pivotal role in many ways including cognitive development, growth retardation, development of chronic diseases later in life, and mortality from common childhood illnesses [1,2,3]. Although all nutrients are necessary for brain development, key nutrients that support neurodevelopment include protein, zinc, iron, choline, folate, iodine, vitamins A, D, B_6_, and B_12_, and long-chain polyunsaturated fatty acids [4]. Also, an excess protein intake during infancy may increase the secretion of insulin and insulin-like growth factor I (IGF-I), thereby promoting weight gain and, in turn, subsequent obesity in later childhood and adolescence [5].

Therefore, understanding the complex interplay between micro- and macronutrients and neurodevelopment and health outcomes is a key factor to moving beyond simply advocating for a generic “healthy diet” and instead focusing on optimizing nutrient intake tailored to the unique needs of infants and toddlers [4].

Recently, in developed countries, there has been a notable surge in dietary modifications, characterized by a growing preference for organic and vegetarian diets or a reduction in salt and sugar intake. This trend reflects an increasing awareness of the link between diet and health outcomes, as well as a desire to adopt more sustainable and environmentally friendly dietary practices [6,7]. Notably, these dietary shifts are not limited to adults but extend to families and parents who are seeking to impart those habits to their children. In this line, novel approaches to infant feeding have emerged, including the baby-led weaning (BLW) method, vegetarian, organic, and vegan feeding guides, increasing the prevalence of breastfeeding, as well as new insights into different types of milk and vegetal beverages, consumption of proteins, or early introduction of potentially allergenic nutrients and food allergy prevention [8,9,10,11,12]. As a result, paediatricians are increasingly being approached by families seeking guidance on adapting their children’s diet to align with vegetarian or other specialized dietary patterns. Despite this, there is consistent scientific evidence of the limitations of these dietary patterns during the complementary feeding period for providing critical concentrations of vitamins and micronutrients, so deficiencies in growth and development should be carefully considered [13]. Data from observational studies also pointed out a potential risk of iron and energy inadequacy as well as the choking risk of BLW [14].

Parents today face an increasing amount of information about parenting available to them online. Overall, dietary practices during the first two years of life are changing in contemporary times and, especially among parents of younger children, the internet constitutes an important and accessible source of information and support about parenting. However, online parenting information may also be overwhelming, confusing, or stressful for parents, leading to less confidence in their parenting [15]. Appropriate, evidence-based nutrition websites should be promoted, and paediatricians should be aware of the need to provide an opportunity for parents to discuss and assess the credibility of the information retrieved [16].

In the nutrition monitoring of children aged birth to 24 months of the National Health and Nutrition Examination Survey (NHANES) of the USA, limited adherence to recommendations of the American Academy of Paediatrics (AAP) for exclusive breastfeeding for about 6 months, solid foods introduction, and vitamin D and iron supplementation was reported [17]. In this survey, however, data related to BLW, types of milk intake during the first 2 years of life, or organic food and vegetarian/vegan diets were not provided as it was not the objective. Moreover, survey studies among paediatricians regarding early nutrition practices have shown that available information on these topics is extremely limited [18,19,20,21].

As paediatricians fulfill a fundamental role in nutritional habits in early childhood, the present study was designed to gather information on the opinions of these health professionals in the Spanish primary care setting regarding different clinically relevant issues in the nutrition of infants during the first 2 years of life. Assessment of their current knowledge and adherence to guidelines and scientific evidence is important to be aware of the quality of the evidence on which decisions about infant nutrition can be based in daily practice and seek to illuminate potential gaps in knowledge and identify areas for improvement in paediatric care practices.

## 2. Materials and Methods

### 2.1. Design and Participants

A cross-sectional survey study (the ACTA project, acronym for Alimentación, Controversias, Tendencias y Actitudes en Pediatria, “Feeding, Controversies, Trends and Attitudes in Paediatrics”) was designed to collect information on clinically relevant topics on feeding patterns in infants during the first 2 years of life of specialists in paediatrics working in the primary care setting throughout Spain. The objectives of the study were to determine the paediatricians’ opinions related to the following 10 clinically relevant topics: (1) exclusive breastfeeding and prevention of allergic disease; (2) the need to boil de water for preparing feeding bottles; (3) Baby-led weaning (BLW); (4) early introduction of potentially allergenic foods and allergy prophylaxis; (5) type of milk recommended during the first 2 years of life; (6) protein consumption during the first 2 years of life; (7) salt intake in the infant diet; (8) sugar intake in the infant diet; (9) vegetarian diet during infancy; and (10) organic foods during infancy.

Candidates to participate in the study were specialists in Paediatrics involved in the care of children who attended public or private consultations throughout Spain. Paediatricians were eligible, provided that they usually take care of a minimum of 10 outpatient infants daily. Participants were recruited through invitations to clinical paediatricians registered in the database of more than 5000 paediatricians, around 50% of the global amount in Spain, of Laboratorios Ordesa, a national pharmaceutical company specializing in infant nutrition and paediatric food supplements. Participation in the study was voluntary and anonymous. The sample was nonrandomized and proportionally stratified to the number of specialists in Paediatrics registered in the autonomous communities of the country. Paediatricians who gave consent to take part in the study were provided with a digital data collection logbook. The fieldwork lasted 12 months and was completed between January and December 2022.

According to the characteristics of the project in which patients did not participate, approval of the study by an Ethics committee was not required. Written informed consent was obtained from all participating paediatricians.

### 2.2. Study Questionnaire and Data Collection

The study questionnaire was developed after a comprehensive literature review in the main databases (PubMed, EMBASE, and Cochrane Controlled Register of Trials [CENTRAL]) using keywords related to the clinically relevant topics identified. The articles of interest were collected and used to develop the study questionnaire, which was reviewed, modified, and approved by two specialists in paediatrics with a large experience in infant nutrition. The questionnaire included between 7 and 11 items for each of the 10 clinically relevant topics identified. The degree of agreement with each question was evaluated using a 5-point Likert scale from 1: strongly disagree, 2: disagree, 3: neither agree or disagree, 4: agree, and 5: strongly agree. The first part of the questionnaire included data regarding demographic characteristics of participants, type of practice, working centre, and number of patients ≤ 2 years of age attended per month. The study questionnaire is described in the Supplementary Materials (Table S1).

### 2.3. Statistical Analysis

Categorical variables are expressed as frequencies and percentages, and quantitative variables as mean and standard deviation (SD) or median and interquartile range (IQR) (25th–75th percentile). The mean (SD) score for responses to each questionnaire item was calculated. The distribution of variables according to the age of participants (categorized as 29–55 years vs. 56–70 years) was analysed using Fisher’s exact test for the comparison of categorical variables, and the Student’s *t* test or the analysis of variance (ANOVA) for the comparison of two or three groups of quantitative variables, respectively, or the Mann–Whitney U test or the Kruskal–Wallis test according to the conditions of application. Statistical significance was set at *p* < 0.05. Statistical Analysis Systems (SAS Institute, Cary, NC, USA) version 9.4 was used for data analysis.

## 3. Results

### 3.1. General Characteristics of Participants

The study population included 245 paediatricians, 54.3% men, and 44.5% women, with a mean (SD) age of 54.7 (9.6) years (range 29–70 years). Within that, 110 paediatricians (44.9%) were aged between 25 and 55 years, and the remaining 135 (55.1%) were between 56 and 70. Most paediatricians (92%) worked in an urban area, 35% in a public centre, 30% in both public and private institutions, and 53% in outpatient hospital consultations. The median number of infants under 2 years of age visited per month was 160 (IQR 8-150). Details of the participants are shown in Table S2 of the Supplementary Materials.

### 3.2. Exclusive Breastfeeding and Prevention of Allergic Diseases

Paediatricians reported to agree or strongly agree with the concept that breastfeeding reduces the risk of allergy in the general population and in infants with a high risk of allergy, particularly, breastfeeding exclusively during the first 6 months of life. Descriptive results are shown in Table 1. Also, 34.7% of the surveyed paediatricians reported insufficient knowledge regarding this topic. Significant differences in responses according to gender and age of participants were not observed. However, the mean ± SD of the item “it is a topic that I do not have enough knowledge about” was 2.1 (1.0) for the younger group (29–55 years) and 1.8 (0.8) for the older group (56–70 years) (*p* = 0.012).

### 3.3. Whether or Not to Boil the Water to Prepare the Feeding Bottle

As shown in Table 2, most paediatricians (between 65.9% and 71.6%) were in favour of recommending boiling water before preparing infants’ formula, and 55.2% expressed concern about using tap water without boiling it before. Also, although most participants recognized they had sufficient knowledge on this subject, they admitted that they would like to learn more about it. Concerning age, the group of younger paediatricians (29–55 years) scored higher in not having enough knowledge (2.8 vs. 2.0, *p* < 0.001), consideration of boiling tap drinking water as an outdated measure (3.0 vs. 2.6, *p* = 0.03), and interest to have more information (3.5 vs. 3.2, *p* = 0.03). The older age group (56–70 years) scored higher in concerns of preparing bottles with tap water without boiling (3.6 vs. 3.2, *p* = 0.005) and recommendation of boiling water when feeding bottles are prepared with tap water (3.8 vs. 3.6, *p* = 0.03).

### 3.4. BLW versus Traditional Complementary Feeding

The results of this section of the questionnaire are shown in Table 3. A 64.3% of paediatricians reported that BLW is a topic frequently asked about by parents. A large majority (82%) advised parents about the risk of choking associated with BLW, 65% were concerned regarding infants receiving an insufficient variety and quantity of nutrients, and 67.2% were in favour of recommending the introduction of traditional pureed complementary feeding. However, 66.4% agreed that BLW improved the transition to solid food by offering varied textures and promoting chewing abilities, and 49.4% considered that BLW may prevent obesity as the infant manages the amount of food to eat and satiety. Only 15.4% agreed or strongly agreed on actively recommending the BLW method in their consultations.

The group of paediatricians aged 29–55 years scored higher in the item that BLW is a topic frequently asked by parents (3.89 vs. 3.56, *p* = 0.01), active recommendation of BLW (2.72 vs. 2.19, *p* = 0.0001), recognition of having insufficient information (2.36 vs. 1.95, *p* = 0.005), and considering that BLW improves psychomotor skills (3.88 vs. 3.42, *p* = 0.001) and transition to solid foods (3.95 vs. 3.60, *p* = 0.007) compared with those aged 56–70 years. The older group scored higher in the recommendation of pureed traditional complementary feeding (4.02 vs. 3.43, *p* < 0.0001) and concern for not receiving sufficient variety and quantity of nutrients (3.86 vs. 3.34, *p* = 0.005).

### 3.5. Early Introduction of Potentially Allergenic Nutrients and Allergy Prevention

As shown in Table 4, the items in which most participants agreed or strongly agreed (77.4%) was the lack of evidence for delaying the introduction of gluten and other allergenic food in the complementary feeding of healthy infants and to delay such introduction in children with a history of asthma, atopy or food allergy (58.9%). On the other hand, many paediatricians disagreed with waiting for 12 months of age for the introduction of eggs o crushed nuts to avoid allergy problems (63.6%) and to delay the introduction of gluten beyond 6 months in infants with a family history of celiac disease (56.8%).

Concerning age groups, younger paediatricians (29–55 years) scored higher in lack of evidence to delay the introduction of potentially allergenic foods (4.1 vs. 3.8, *p* = 0 0.006), insufficient knowledge (2.5 vs. 2.2, *p* = 0.002), and interest to have more information (3.5 vs. 3.2, *p* = 0.015), whereas older paediatricians (56–70 years) scored higher in the delay of the introduction of potentially allergenic foods in children with a history of asthma, atopy, or food allergy (3.7 vs. 3.2, *p* = 0.0003), waiting until 1 year of age to introduce eggs or crushed nuts (2.7 vs. 2.0, *p* < 0.001), and delaying the introduction of gluten beyond 6 months in children with a family history of celiac disease (2.8 vs. 2.4, *p* = 0.027).

### 3.6. Type of Milk Recommended during the First Two Years of Life

In this section of the questionnaire, 84.9% of participants agreed or strongly agreed that two servings of dairy products (equivalent to two glasses of 200 mL of cow’s milk) is the adequate amount in a diversified diet and 74.8% that cow’s milk should not be introduced as the main source of dairy foods before 12 months. Additionally, 62.9% considered that the exclusive use of vegetarian beverages in young children entails health risks. Moreover, 93.4% disagreed or strongly disagreed with the use of vegetal beverages in infants <1 year old instead of cow’s milk. Results are shown in Table 5.

Paediatricians in the younger age group showed higher scores in lack of sufficient knowledge (2.56 vs. 2.12, *p* = 0.0003), whereas those in the older group showed higher scores in recommending skimmed milk in overweight infants or with family risk factors (2.94 vs. 2.26, *p* = 0.0001) and agreed with the recommendation of two servings of dairy products a day (equivalent to two glasses of 200 mL of cow’s milk) (4.21 vs. 3.79, *p* = 0.0002).

### 3.7. Protein Consumption during the First Two Years of Life

Concerning protein consumption in the infant’s diet, 86.7% of paediatricians considered that it is not necessary to include meat every day in the menu, 66.4% that excessive protein intake at an early age increases the risk of obesity in the future, 65.5% advise to primarily consume white meats, and 60.6% that children from 6 months to 2 years old consume too much protein in their daily diet. Descriptive results and mean scores for each item are shown in Table 6.

The analysis of this section of the questionnaire by age showed higher mean scores in the younger age group (29–55 years) for insufficient knowledge (2.6 vs. 2.1, *p* < 0.001), the idea that an excess of protein consumption at early age increases the risk of obesity (4.0 vs. 3.5, *p* < 0.001), and the need to have more information (3.6 vs. 3.2, *p* = 0.07). However, the older age group (56–70 years) scored higher in preference for consumption of proteins of vegetal origin (3.4 vs. 2.9, *p* < 0.001), the consideration that parents are concerned about insufficient consumption of proteins (3.4 vs. 3.2, *p* = 0.045), and the agreement that an increase in protein intake in school-age children with high physical activity is needed (3.2 vs. 3.0, *p* = 0.022).

### 3.8. Salt Intake in the Diet during the First Two Years of Life

93.8% of paediatricians considered that before one year of life, it is not advisable to add salt to foods during the complementary feeding period, 88.4% that after the age of one, it is advisable to cook with a little bit of salt, preferably iodized one, and 82.2% that excessive sodium intake in early childhood may program the development of higher blood pressure in later stages of life (see Table 7).

Younger paediatricians (29–55 years) scored higher than the older group (56–70 years) in the opinion that it is not advisable to add salt during complementary feeding (4.6 vs. 4.4, *p* = 0.021), insufficient knowledge of this topic (2.5 vs. 2.1, *p* = 0.003), and interest to have more information (3.3 vs. 3.1, *p* = 0.023). On the other hand, older paediatricians scored higher in adding salt to increase the taste of meals to make children eat better (1.7 vs. 1.4, *p* = 0.013).

### 3.9. Sugar Intake in the Diet during the First Two Years of Life

In this section of the questionnaire (Table 8), most paediatricians agreed or strongly agreed with all items in which the negative aspects of sugar consumption at an early age were emphasized (see Table 8), particularly obesity in childhood (87.9%), alteration of food taste perception (79.6%), and avoidance of any added sugar intake during the first two years of life (75.7%). Moreover, 66.4% believed that regular and continued consumption of sugar in early childhood can be a cause of diabetes in the future.

Differences according to the paediatrician’s age showed higher mean scores in the younger group (29–55 years) for the continuous and regular use of sugar at an early age as a cause of diabetes in the future (4.0 vs. 3.6, *p* = 0.008) and can alter the taste perception (4.1 vs. 3.9, *p* = 0.045). Younger paediatricians also were more interested in having more information (3.5 vs. 3.1, *p* = 0.009), whereas the older group (56–70 years) scored higher in recommending an increased sugar intake for school-age children who engage in high physical activity (2.0 vs. 1.7, *p* = 0.018).

### 3.10. Vegetarian Diet during the First Two Years of Life

More than 65% of paediatricians expressed concerns about several aspects of vegetarian diets in infants and toddlers (Table 9). They recommended supplementation of vegetarian diets of their patients with vitamin B_12_ (73.4% of participants), iron (67.9%), and vitamin D (66.2%). Furthermore, 73.8% agreed with the risk of anaemia and vitamin B_12_ deficiency associated with vegetarian diets during the first two years of life.

In the comparison of responses to this section of the questionnaire, paediatricians from the younger age category rated higher than those in the older group in items like that an appropriately planned vegetarian diet is healthy, nutritionally adequate, and can provide health benefits in the prevention and treatment of certain diseases (2.75 vs. 2.38, *p* = 0.013), insufficient knowledge on this topic (3.15 vs. 2.65, *p* = 0.0003) and recommendation to supplement vegetarian diets with vitamin D (3.89 vs. 3.43, *p* = 0.003).

### 3.11. Organic Foods during the First Two Years of Life

Regarding organic foods, 55% of paediatricians considered that it is not necessary to buy organic foods for children, 42.1% agreed with the idea that organic and non-organic foods have similar nutritional properties, and only 36.7% preferred that their patients consume organic foods. However, as is shown in Table 10, the option ‘neither agree nor disagree’ was selected for all items by more than one-third of paediatricians. Also, 70.1% of paediatricians did not agree with the proposal that organic foods have a better nutritional profile than non-organic foods.

Regarding the age categories, younger paediatricians scored higher in the item of having insufficient knowledge on this topic as compared to the older group (3.05 vs. 2.60, *p* = 0.0005).

## 4. Discussion

The present study presents data on the opinion of primary care paediatricians regarding several clinically relevant topics related to infant and toddler nutrition during the first two years of life based on their experience in daily practice conditions. The study population of 245 specialists in paediatrics included a broad number of health professionals from across the country and there was a representation of all autonomous communities. Paediatricians’ median age of around 56 years was indicative of the notable experience that they have accumulated in their professional activity. Distribution by age groups was similar as well as by the type of centre (public or private).

The design of the study questionnaire was based on an extensive review of the literature and the 10 topics included with their corresponding items were selected according to currently clinically relevant topics in infant nutrition, highlighting as common current topics the advantages and disadvantages of BLW, vegetarian diets, and organic foods.

### 4.1. Breastfeeding, Formula Feeding, and Milk Choices under Two Years of Life

Scientific societies and the World Health Organization (WHO) actively advocate for breastfeeding as the optimal method for infant feeding, recommending exclusive breastfeeding for the first six months of life. The relevance of breastfeeding as an exclusive source of early nutrition from birth to at least 6 months of age was also recognized by the surveyed paediatricians, with the clinical significance of providing protection and lowering the risk of respiratory tract infections, diarrhoea, and developing asthma, rhinitis, type 1 diabetes, food allergies, and obesity as previous publications had demonstrated in different clinical trials [22,23,24]. On the other hand, concerning bottle-fed infants, within our research, paediatricians’ opinions supported recommending boiling water before preparing infant’s feeding bottles in concordance with the studies that had shown that combination of handwashing, use of safe (tap) water, and boiling improve the microbiological safety of infant formula feeding [25,26].

Regarding milk intake, the main point of interest was the high rate of agreement with the statement that established that consumption of two dairy servings in a diversified diet between 1 and 3 years of age was the most adequate option, following scientific recommendations either European or American [27,28].

Likewise, most of the respondents agreed with the fact that cow’s milk should not be introduced as the main source of dairy before 12 months of age as well as with the health risks that entail the exclusive use of vegetarian beverages in infants younger than 1 year old. Plant-based beverages are gaining prominence among consumers in many developed countries. Although many of them attempt to optimize the micronutrient profile compared to cow’s milk, they usually contain high levels of added sugars to enhance organoleptic characteristics worsening their theoretical nutritional advantages [29]. In general terms, in this survey, health professionals agreed with the consensus that plant-based beverages should be avoided during the first two years of life and that in the first 12 months of age, the optimal beverage for infants’ growth is breast milk, with infant formulas used when are needed [30]. In our study, most paediatricians acknowledged that they would like to have more information on this topic and that parents frequently asked them for the most convenient type of milk in the initial two years of infancy.

### 4.2. Complementary Foods: Baby-Led Weaning (BLW) vs. Traditional Diversification

Regarding the topic of the BLW method, most paediatricians were concerned with the idea that infants might receive an insufficient variety and quantity of nutrients and thus up to 67% of the respondents were in favour of recommending the introduction of traditional pureed complementary feeding, although recognizing that BLW may improve the acceptability of food textures and chewing abilities as well as promoting better satiety responsiveness. However, most paediatricians also advise on the risk of choking associated with BLW. These have been documented in the literature, emphasizing the relevance of introducing iron- and energy-rich complementary foods and avoiding foods that may present choking hazards [31].

Therefore, as we exposed in the results of our study, only a low percentage of participants (15.4%) actively recommended BLW in their consultations. In comparison, in another retrospective survey study of 38 Spanish healthcare professionals (doctors, nurses, and midwives) who provided hospital paediatric care in a region of Leon (Spain), 15.8% of the respondents will never recommend BLW, 57.9% sometimes and 26.3% always [32].

Despite the advantages and increasing popularity of the BLW method, there is not enough scientific evidence to conclude the BLW approach [33,34,35]. In our study, 48.7% of paediatricians would like to have more information on BLW practices. In this line, other studies have shown that parents’ knowledge of the BLW method is insufficient [36]. Review studies of the BLW method have concluded that, despite the benefits, longitudinal randomized controlled studies are needed to ensure the safety of the method when practiced exclusively [14,33]. Studies in this field should be conducted as the introduction of complementary foods is crucial and not easy. Nutrition during the weaning period is usually progressive and leads the infant to reach the dietary pattern of an adult within the second year of life. The weaning period is a crucial time in an infant’s life since it not only involves a great deal of rapid change for the child but is also associated with the development of food preferences and eating behaviours that influence childhood, adolescence, and adulthood as well and, therefore, it is necessary to provide evidence-based advice to parents.

### 4.3. Protein, Salt and Sugar

In the sections on protein, salt, and sugar content in the diet, there was a general agreement on the need to avoid salt and added sugar according to general recommendations for healthy nutrition during the first two years of life, preventing negative side effects of their excessed intake and sugar caloric contribution [37,38,39,40]. Excessive consumption of these ingredients has been linked to several adverse health outcomes including obesity, cardiovascular disease, and hypertension. Their prevalence in commercial toddler meals, cereal bars, breakfast pastries, and infant/toddler snacks and desserts poses significant health risks for children. That is why it is imperative to support parents in making informed dietary choices for their infants and toddlers. By encouraging parents to reduce consumption of these products and choose healthier alternatives, the promotion of long-term health and well-being can be improved, and the negative impact of poor dietary habits can be mitigated.

### 4.4. Vegetarian Diets

Concerning vegetarian diets, the study findings point out a high percentage of participants’ concerns regarding the limitations of plant-based diets for adequate provision of vitamins B_12_, D, and iron. However, it has been argued that well-planned vegan diets using supplementation are likely to provide the recommended amounts of critical nutrients [41]. By contrast, there are reviews in the literature on the potential outcomes of vegetarian diets during complementary feeding that conclude that these diets cannot be actively recommended due to their potential side effects caused by vitamin and micronutrient deficiencies on growth and development [13,42].

### 4.5. Organic Foods

Regarding organic foods, almost 70% of the paediatricians who participated in our study disagreed with the statement that they have a better nutritional profile than non-organic ones. Along the same line, over half of the paediatricians considered that buying organic foods is not necessary, taking into account that they may be more expensive. Related to organic foods, an analysis made in France with more than 8000 children concluded the use of organic products during complementary feeding was associated with higher education levels of the mothers and family income [43] but consuming them during complementary feeding was not associated with reduced odds of food allergy later in childhood [44].

An interesting finding of the study regarding differences by age was the greater interest of younger paediatricians in recognizing the lack of sufficient knowledge and the interest in receiving more information on many of the topics presented, in particular the introduction of potentially allergenic foods, protein consumption and the risk of obesity, salt and sugar intake, and vegetarian diet.

Limitations of the present study include selection bias possibly affecting the representativeness of the sample and generalizability of the study findings. However, it presents several strengths as the size of the sample, the wide diversity of nutrition-related topics in infants and toddlers during the first two years of life, especially those related to the BLW method, types of milk, vegetarian diets, and organic foods.

An interesting finding in the assessment of differences by participants’ age, was the greater interest of younger paediatricians in recognizing the lack of sufficient knowledge and the interest in receiving more information on many of the topics presented, in particular the introduction of potentially allergenic foods, high protein consumption and the risk of obesity, risks of high salt and sugar intake, and vegetarian diet. These results support the need for specific actions, such as workshops, seminars, or monographic courses aimed to improve the training of young paediatricians in these areas.

Furthermore, the authors consider that identifying these nutrition-related clinically relevant issues, and training paediatricians about them, is fundamental. Passing all this knowledge, especially to young parents who mainly seek online information that can be overwhelming and confusing, would lead to more confident parenting [15].

## 5. Conclusions

The present study provides a current perspective of a large sample of experienced Spanish paediatricians opinions regarding some clinically relevant aspects related to children feeding up to the age of 2 years. The most relevant findings were as follows:

- To advise parents about the risk of choking associated with BLW and concerns regarding insufficient variety and quantity of nutrients with this practice.
- Lack of evidence for delaying the introduction of gluten and other allergenic food in the complementary feeding of healthy infants.
- Two servings of dairy (equivalent to two glasses of 200 mL of cow’s milk) is the recommended amount in a diversified diet.
- To boil water before preparing the infant´s feeding bottles.
- Disagreement on the use of vegetal beverages in infants younger than 1 year old instead of cow’s milk.
- To avoid added salt and sugar.
- Organic foods do not have a better nutritional profile than non-organic foods.
- Limitations of vegetarian diets especially for adequate provision of vitamins B_12_, D, and iron.

In general, there is adequate knowledge of new trends, which prompts many questions from parents and generates interest among professionals seeking more information. The desire to obtain additional training was higher among younger pediatricians and, consequently, health professionals societies and expert groups can play an important role in improving knowledge on these topics including them in their scientific activities.

## Figures and Tables

**Table 1:** Paediatricians’ opinions regarding exclusive breastfeeding and allergy prevention.

Questions	*N*	5—Point Likert Scale, *n* (%)					Mean SD
		1	2	3	4	5	
It is a topic that parents frequently ask me about	243	13 (5.3)	39 (15.6)	57 (23.5)	68 (28.0)	67 (27.6)	3.6 (1.2)
In all children, breastfeeding reduces the risk of allergies, especially if exclusive breastfeeding is given during the first 6 months of life	244	5 (2.0)	27 (11.1)	37 (15.2)	89 (36.5)	86 (35.2)	3.9 (1.1)
In children at a high risk of allergy due to genetic history/causes, breastfeeding reduces the risk of allergy, especially if during the first 6 months it is given exclusively	243	3 (1.2)	16 (6.6)	29 (11.9)	98 (40.3)	97 (39.9)	4.1 (0.9)
It is a topic that I do not have enough knowledge about	239	83 (34.7)	106 (44.4)	30 (12.6)	17 (7.19)	3 (1.3)	20.0 (0.9)
Exclusive breastfeeding does not confer protection for the prevention of allergic diseases	241	98 (40.7)	69 (28.6)	33 (13.7)	34 (14.1)	7 (2.9)	2.1 (1.2)
It is advisable to avoid exposure to small amounts of cow’s milk (infant formulas) during the first days of the infant’s life as they appear to increase allergy to cow’s milk protein	241	32 (13.3)	47 (19.5)	35 (14.5)	66 (27.4)	61 (25.3)	3.3 (1.4)
I recommend to families of my patients to extend exclusive breastfeeding as much as possible to protect their babies from the risk of allergies	244	12 (4.9)	35 (14.3)	66 (27.0)	87 (35.7)	44 (18.0)	3.5 (1.1)
This is a topic I would like to have more information on	239	12 (5.0)	8 (3.3)	66 (27.6)	109 (45.6)	44 (18.4)	3.7 (1.0)

*N*: number of participants; 1: strongly disagree, 2: disagree, 3: neither agree nor disagree, 4: agree, and 5: strongly agree; SD: standard deviation.

**Table 2:** Paediatricians’ opinions regarding the need to boil the water for preparing feeding bottles.

Questions	*N*	5—Point Likert Scale, *n* (%)					Mean SD
		1	2	3	4	5	
It is a topic that parents frequently ask me about	241	19 (7.9)	43 (17.8)	46 (19.1)	100 (41.5)	33 (13.7)	3.4 (1.2)
If public tap water only undergoes chlorination or rapid filtration, as is the case in many municipalities, it should be boiled to inactivate any cysts or oocysts it may contain	240	6 (2.5)	21 (8.8)	41 (17.1)	89 (37.1)	83 (34.6)	3.9 (1.0)
The boiling duration should be only 1 min to avoid the added risk of excessive ionic input	240	7 (2.9)	20 (8.3)	50 (20.8)	87 (36.3)	76 (31.7)	3.9 (1.1)
It is a topic that I do not have enough knowledge about	234	65 (27.8)	64 (27.4)	62 (26.5)	33 (14.1)	10 (4.3)	2.4 (1.2)
I am concerned that parents prepare their children’s feeding bottles with water for public consumption without boiling it first	239	21 (8.8)	46 (19.2)	40 (16.7)	83 (34.7)	49 (20.5)	3.4 (1.3)
I recommend to families of my patients that, if they prepare the feeding bottles with tap water for public consumption, they must always boil it before	238	13 (5.5)	36 (15.1)	32 (13.4)	83 (34.9)	74 (31.1)	3.7 (1.2)
I consider that currently in our country this measure (boiling tap drinking water before preparing the baby feeding bottle) is outdated	241	47 (19.5)	52 (21.6)	64 (26.6)	57 (23.7)	21 (8.7)	2.8 (1.2)
This is a topic I would like to have more information on	238	23 (9.7)	27 (11.3)	72 (30.3)	77 (32.4)	39 (16.4)	3.3 (1.2)

*N*: number of participants; 1: strongly disagree, 2: disagree, 3: neither agree nor disagree, 4: agree, and 5: strongly agree; SD: standard deviation.

**Table 3:** Paediatricians’ opinions regarding BLW versus traditional complementary feeding.

Questions	*N*	5—Point Likert Scale, *n* (%)					Mean SD
		1	2	3	4	5	
It is a topic that parents frequently ask me about	241	4 (1.7)	33 (13.7)	49 (20.3)	97 (40.2)	58 (24.1)	3.7 (1.0)
I actively recommend the practice of BLW in my consultation	240	59 (24.6)	60 (25.0)	84 (35.0)	29 (12.1)	8 (3.3)	2.4 (1.1)
I advise parents to introduce traditional pureed complementary feeding	241	7 (2.9)	19 (7.9)	53 (22.0)	116 (48.1)	46 (19.1)	3.7 (1.0)
It is a topic that I do not have enough knowledge about	236	74 (31.4)	87 (36.9)	45 (19.1)	25 (10.6)	5 (2.1)	2.2 (1.0)
I am concerned that an infant practicing BLW may not receive a sufficient variety and quantity of nutrients	240	14 (5.8)	40 (16.7)	30 (12.5)	98 (40.8)	58 (24.2)	3.6 (1.2)
I warn parents about the risk of choking associated with BLW	239	6 (2.5)	9 (3.8)	28 (11.7)	100 (41.8)	96 (40.2)	4.1 (0.9)
The BLW encourages and promotes the psychomotor development of infants, allowing them to develop the different skills they will need to eat	240	10 (4.2)	18 (7.5)	76 (31.7)	79 (32.9)	57 (23.8)	3.6 (1.1)
BLW improves the transition to solid food by offering varied textures and promoting chewing abilities	241	8 (3.3)	18 (7.5)	55 (22.8)	100 (41.5)	60 (24.9)	3.8 (1.0)
BLW prevents obesity because the infants who manage the amount of food they eat and the sensations of satiety	241	18 (7.5)	32 (13.3)	72 (29.9)	68 (28.2)	51 (21.2)	3.4 (1.2)
Most of the families in my consultation use BLW to introduce complementary feeding	240	52 (21.7)	101 (42.1)	68 (28.3)	14 (5.8)	5 (2.1)	2.2 (0.9)
This is a topic I would like to have more information on	236	29 (12.3)	30 (12.7)	62 (26.3)	77 (32.6)	38 (16.1)	3.3 (1.2)

*N*: number of participants; 1: strongly disagree, 2: disagree, 3: neither agree nor disagree, 4: agree, and 5: strongly agree; SD: standard deviation.

**Table 4:** Paediatricians’ opinions regarding early introduction of potentially allergenic nutrients.

Questions	*N*	5—Point Likert Scale, *n* (%)					Mean SD
		1	2	3	4	5	
It is a topic that parents frequently ask me about	243	13 (5.3)	23 (9.5)	57 (23.5)	111 (45.7)	39 (16.0)	3.6 (1.0)
Currently, no evidence justifies delaying the introduction of potentially allergic foods in the introduction of complementary feeding in healthy children	243	9 (3.7)	16 (6.6)	30 (12.3)	109 (44.9)	79 (32.5)	4.0 (1.0)
It only makes sense to delay the introduction of potentially allergic foods in the introduction of complementary feeding in children with a history of asthma, atopy, or food allergy	241	16 (6.6)	42 (17.4)	41 (17.0)	98 (40.7)	44 (18.3)	3.5 (1.2)
It is a topic that I do not have enough knowledge about	237	43 (18.1)	92 (38.8)	74 (31.2)	21 (8.9)	7 (3.0)	2.4 (1.0)
I prefer to wait until my patients are 1 year old to introduce foods such as eggs or crushed nuts to avoid allergy problems	239	77 (32.2)	75 (31.4)	26 (10.9)	39 (16.3)	22 (9.2)	2.4 (1.3)
In children with a family history of celiac disease, I recommend delaying the introduction of gluten into their diet beyond 6 months	241	66 (27.4)	71 (29.5)	24 (10.0)	54 (22.4)	26 (10.8)	2.6 (1.4)
This is a topic I would like to have more information on	237	17 (7.2)	39 (16.5)	63 (26.6)	80 (33.8)	38 (16.0)	3.4 (1.1)

*N*: number of participants; 1: strongly disagree, 2: disagree, 3: neither agree nor disagree, 4: agree, and 5: strongly agree; SD: standard deviation.

**Table 5:** Paediatricians’ opinions regarding the type of milk during the first two years of life.

Questions	*N*	5—Point Likert Scale, *n* (%)					Mean SD
		1	2	3	4	5	
It is a topic that parents frequently ask me about	240	20 (8.3)	26 (10.8)	34 (14.2)	107 (44.6)	53 (22.1)	3.6 (1.2)
Cow’s milk should not be introduced as the main source of dairy foods before 12 months of life, although small amounts can be added earlier	239	16 (6.7)	25 (10.5)	19 (7.9)	86 (36.0)	93 (38.9)	3.9 (1.2)
The exclusive use of non-infant vegetable beverages in young children (1–3 years old) entails serious health risks	240	9 (3.8)	33 (13.8)	47 (19.6)	74 (30.8)	77 (32.1)	3.7 (1.2)
It is a topic that I do not have enough knowledge about	236	50 (21.2)	83 (35.2)	77 (32.6)	22 (9.3)	4 (1.7)	2.4 (1.0)
I consider that there are no significant differences in the growth and development of children fed with formula made from goat’s milk or cow’s milk, so their nutritional qualities can be considered similar	238	19 (8.0)	38 (16.0)	72 (30.3)	84 (35.3)	25 (10.5)	3.2 (1.1)
I prefer that young children (>1 year old) consume vegetable beverages (oats, almonds, rice, coconut…) instead of cow’s milk	242	163 (67.4)	63 (26.0)	11 (4.5)	2 (0.8)	3 (1.2)	1.4 (0.7)
The recommended reasonable amount in a diversified and complete diet for a child aged 1 to 3 years should be two servings of dairy products (equivalent to 2 glasses of 200 mL of cow’s milk)	230	3 (1.3)	16 (6.7)	17 (7.1)	143 (59.8)	60 (25.1)	4.0 (0.8)
I recommend switching to skimmed milk for children between 12 months and 2 years old who are overweight or have family members with high cholesterol levels or other health-related risk factors	241	61 (25.3)	67 (27.8)	36 (14.9)	56 (23.2)	21 (8.7)	2.6 (1.3)
This is a topic I would like to have more information on	241	61 (25.3)	32 (13.3	61 (25.3)	82 (34.0)	46 (19.1)	3.4 (1.2)

*N*: number of participants; 1: strongly disagree, 2: disagree, 3: neither agree nor disagree, 4: agree, and 5: strongly agree; SD: standard deviation.

**Table 6:** Paediatricians’ opinions regarding protein consumption during the first two years of life.

Questions	*N*	5—Point Likert Scale, *n* (%)					Mean SD
		1	2	3	4	5	
It is a topic that parents frequently ask me about	239	18 (7.5)	72 (30.1)	73 (30.5)	52 (21.8)	24 (10.0)	3.0 (1.1)
I believe that currently, children from 6 months to 2 years old consume too much protein in their daily diet	241	8 (3.3)	44 (18.3)	43 (17.8)	110 (45.6)	36 (14.9)	3.5 (1.1)
In children from 6 months to 2 years, it is preferable to consume protein of animal origin rather than plant origin	241	10 (4.1)	59 (24.5)	78 (32.4)	74 (30.7)	20 (8.3)	3.1 (1.0)
Children’s menus do not need to include meat every day, as it can be alternated with other protein-rich food groups	241	7 (2.9)	10 (4.1)	15 (6.2)	127 (52.7)	82 (34.0)	4.1 (0.9)
Parents are concerned that children may not consume enough proteins	242	9 (3.7)	52 (21.5)	66 (27.3)	84 (34.7)	31 (12.8)	3.3 (1.1)
It is a topic that I do not have enough knowledge about	238	56 (23.5)	78 (32.8)	77 (32.4)	22 (9.2)	5 (2.1)	2.3 (1.0)
I regularly advise that children primarily consume white meats due to their lower fat content	241	14 (5.8)	25 (10.4)	44 (18.3)	118 (49.0)	40 (16.6)	3.6 (1.1)
Excessive protein intake at an early age increases the risk of obesity in the future	238	10 (4.2)	29 (12.2)	41 (17.2)	80 (33.6)	78 (32.8)	3.8 (1.2)
I recommend an increase in protein intake for school-age children with high physical activity levels	236	11 (4.7)	46 (19.5)	89 (37.7)	83 (35.2)	7 (3.0	3.1 (0.9)
This is a topic I would like to have more information on	237	19 (8.0)	33 (13.9)	52 (21.9)	98 (41.4)	35 (14.8)	3.4 (1.1)

*N*: number of participants; 1: strongly disagree, 2: disagree, 3: neither agree nor disagree, 4: agree, and 5: strongly agree; SD: standard deviation.

**Table 7:** Paediatricians’ opinions regarding salt intake during the first two years of life.

Questions	*N*	5—Point Likert Scale, *n* (%)					Mean SD
		1	2	3	4	5	
It is a topic that parents frequently ask me about	242	21 (8.7)	39 (16.1)	56 (23.1)	92 (38.0)	34 (14.0)	3.3 (1.2)
Before one year of life, it is not advisable to add salt to foods during the complementary feeding period	241	3 (1.2)	2 (0.8)	10 (4.1)	84 (34.9)	142 (58.9)	4.5 (0.7)
After the age of one, it is advisable to cook with a little bit of salt, preferably iodized one	241	5 (2.1)	6 (2.5)	17 (7.1)	113 (46.9)	100 (41.5)	4.2 (0.8)
It is a topic that I do not have enough knowledge about	236	55 (23.3)	86 (36.4)	72 (30.5)	17 (7.2)	6 (2.5)	2.3 (1.0)
I usually suggest to families to add salt to children’s meals so that they are tastier, and they eat better, regardless of age	242	145 (59.9)	68 (28.1)	17 (7.0)	9 (3.7)	3 (1.2)	1.6 (0.9)
Excessive sodium intake in early childhood may cause the development of higher blood pressure in later stages of life	241	5 (2.1)	7 (2.9)	31 (12.9)	111 (46.1)	87 (36.1)	4.1 (0.9)
This is a topic I would like to have more information on	237	26 (11.0)	38 (16.0)	59 (24.9)	80 (33.8)	34 (14.3)	3.2 (1.2)

*N*: number of participants; 1: strongly disagree, 2: disagree, 3: neither agree nor disagree, 4: agree, and 5: strongly agree; SD: standard deviation.

**Table 8:** Paediatricians’ opinions regarding sugar intake during the first two years of life.

Questions	*N*	5—Point Likert Scale, *n* (%)					Mean SD
		1	2	3	4	5	
It is a topic that parents frequently ask me about	240	5 (2.1)	32 (13.3)	43 (17.9)	109 (45.4)	51 (21.3)	3.7 (1.0)
Children’s sugar consumption is one of the most common concerns among parents	239	5 (2.1)	32 (13.4)	48 (20.1)	108 (45.2)	46 (19.2)	3.7 (1.0)
Until children reach 2 years old, they should not consume any type of added sugar	239	4 (1.7)	17 (7.1)	37 (15.5)	90 (37.7)	91 (38.1)	4.0 (1.0)
Regular and continued consumption of sugar in early childhood can be a cause of diabetes in the future	238	4 (1.7)	27 (11.3)	49 (20.6)	92 (38.7)	66 (27.7)	3.8 (1.0)
It is a topic that I do not have enough knowledge about	235	45 (19.1)	74 (31.5)	83 (35.3)	30 (12.8)	3 (1.3)	2.5 (1.0)
Regular and continued consumption of sugar in early childhood can alter food taste perception in children	240	4 (1.7)	14 (5.8)	31 (12.9)	114 (47.5)	77 (32.1)	4.0 (0.9)
I believe that a large part of the obesity problems in childhood could be avoided by moderating sugar consumption in early ages	239	3 (1.3)	4 (1.7)	22 (9.2)	111 (46.4)	99 (41.4)	4.3 (0.8)
I recommend an increased sugar intake for school-age children who engage in high physical activity	241	111 (46.1)	74 (30.7)	42 (17.4)	10 (4.1)	4 (1.7)	1.8 (1.0)
This is a topic I would like to have more information on	236	24 (10.2)	29 (12.3)	65 (27.5)	84 (35.6)	34 (14.4)	3.3 (1.2)

*N*: number of participants; 1: strongly disagree, 2: disagree, 3: neither agree nor disagree, 4: agree, and 5: strongly agree; SD: standard deviation.

**Table 9:** Paediatricians’ opinions regarding vegetarian diets during the first two years of life.

Questions	*N*	5—Point Likert Scale, *n* (%)					Mean SD
		1	2	3	4	5	
It is a topic that parents frequently ask me about	240	46 (19.2)	83 (34.6)	71 (29.6)	27 (11.3)	13 (5.4)	2.5 (1.1)
In children between 6 months and 2 years, appropriately planned vegetarian diets are healthy, nutritionally adequate, and can provide health benefits in the prevention and treatment of certain diseases	241	41 (17.0)	87 (36.1)	62 (25.7)	38 (15.8)	13 (5.4)	2.6 (1.1)
I consider it nutritionally risky to subject children between 6 months and 2 years to vegetarian diets due to the high risk of anemia and vitamin B_12_ deficiency	241	8 (3.3)	25 (10.4)	30 (12.4)	99 (41.1)	79 (32.8)	3.9 (1.1)
A diet without foods of animal origin cannot meet the nutritional and energy needs of 6-month-old children to 2 years	239	6 (2.5)	46 (19.2)	52 (21.8)	90 (37.7)	45 (18.8)	3.5 (1.1)
It is a topic that I do not have enough knowledge about	238	25 (10.5)	60 (25.2)	86 (36.1)	50 (21.0)	17 (7.1)	2.9 (1.1)
My vegetarian patients have better overall health than patients on an omnivorous diet	238	65 (27.3)	98 (41.2)	69 (29.0)	6 (2.5)	-	2.1 (0.8)
I recommend supplementing the diet of my vegetarian patients (6 months–2 years) with vitamin B_12_	237	4 (1.7)	5 (2.1)	47 (19.8)	102 (43.0)	79 (33.3)	4.0 (0.9)
I recommend supplementing the diet of my vegetarian patients (6 months–2 years) with iron	237	4 (1.7)	17 (7.2)	55 (23.2)	106 (44.7)	55 (23.2)	3.8 (0.9)
I recommend supplementing the diet of my vegetarian patients (6 months–2 years) with vitamin D	237	4 (1.7)	13 (5.5)	63 (26.6)	102 (43.0)	55 (23.2)	3.8 (0.9)
This is a topic I would like to have more information on	238	18 (7.6)	19 (8.0)	54 (22.7)	86 (36.1)	61 (25.6)	3.6 (1.2)

*N*: number of participants; 1: strongly disagree, 2: disagree, 3: neither agree nor disagree, 4: agree, and 5: strongly agree; SD: standard deviation.

**Table 10:** Paediatricians’ opinions regarding organic foods during the first two years of life.

Questions	*N*	5—Point Likert Scale, *n* (%)					Mean SD
		1	2	3	4	5	
It is a topic that parents frequently ask me about	242	44 (18.2)	48 (19.8)	70 (28.9)	61 (25.2)	19 (7.9)	2.8 (1.2)
I prefer that my patients from 6 months to 2 years consume organic foods	240	24 (10.0)	36 (15.0)	92 (38.3)	72 (30.0)	16 (6.7)	3.1 (1.1)
I believe that organic foods are better because they have a superior nutritional profile	241	28 (11.6)	47 (19.5)	94 (39.0)	59 (24.5)	13 (5.4)	2.9 (1.1)
I think organic foods are a fashion and their consumption does not impact the health of my patients	242	15 (6.2)	56 (23.1)	85 (35.1)	62 (25.6)	24 (9.9)	3.1 (1.1)
It is a topic that I do not have enough knowledge about	237	22 (9.3)	69 (29.1)	87 (36.7)	48 (20.3)	11 (4.6)	2.8 (1.0)
Nutritional properties of organic foods are the same as those of nonorganic foods	240	8 (3.3)	40 (16.7)	61 (25.4)	90 (37.5)	41 (17.1)	3.5 (1.1)
Parents who buy organic food for their children do it because they believe that it makes them, in a certain way, better parents	240	9 (3.8)	38 (15.8)	62 (25.8)	94 (39.2)	37 (15.4)	3.5 (1.1)
I tell families that it is not necessary to buy organic foods for their children	240	13 (5.4)	43 (17.9)	102 (42)	60 (25.0)	22 (9.2)	3.1 (1.0)
This is a topic I would like to have more information on	235	14 (6.0)	28 (11.9)	59 (25.1)	85 (36.2)	49 (20.9)	3.5 (1.1)

*N*: number of participants; 1: strongly disagree, 2: disagree, 3: neither agree nor disagree, 4: agree, and 5: strongly agree; SD: standard deviation.

## Data Availability

The data presented in this study are available on request from the corresponding author due to privacy and ethical reasons.

## Supplementary Materials

The following supporting information can be downloaded at: https://www.mdpi.com/article/10.3390/nu16132146/s1, Supplementary material Table S1 Acta study: Study questionnaire. Supplementary material Table S2: General characteristics of participants.
