# Supplementary material for: Clinically Relevant Topics and New Tendencies in Childhood Nutrition during the First 2 Years of Life: A Survey among Primary Care Spanish Paediatricians
Source: Nutrients. 2024 Jul 5;16(13):2146. doi: 10.3390/nu16132146 (PMC11243411; doi:10.3390/nu16132146)
Supplement: Supplementary file 1 [file nutrients-16-02146-s001.zip › Supplementary material Table S1 ACTA study.pdf]

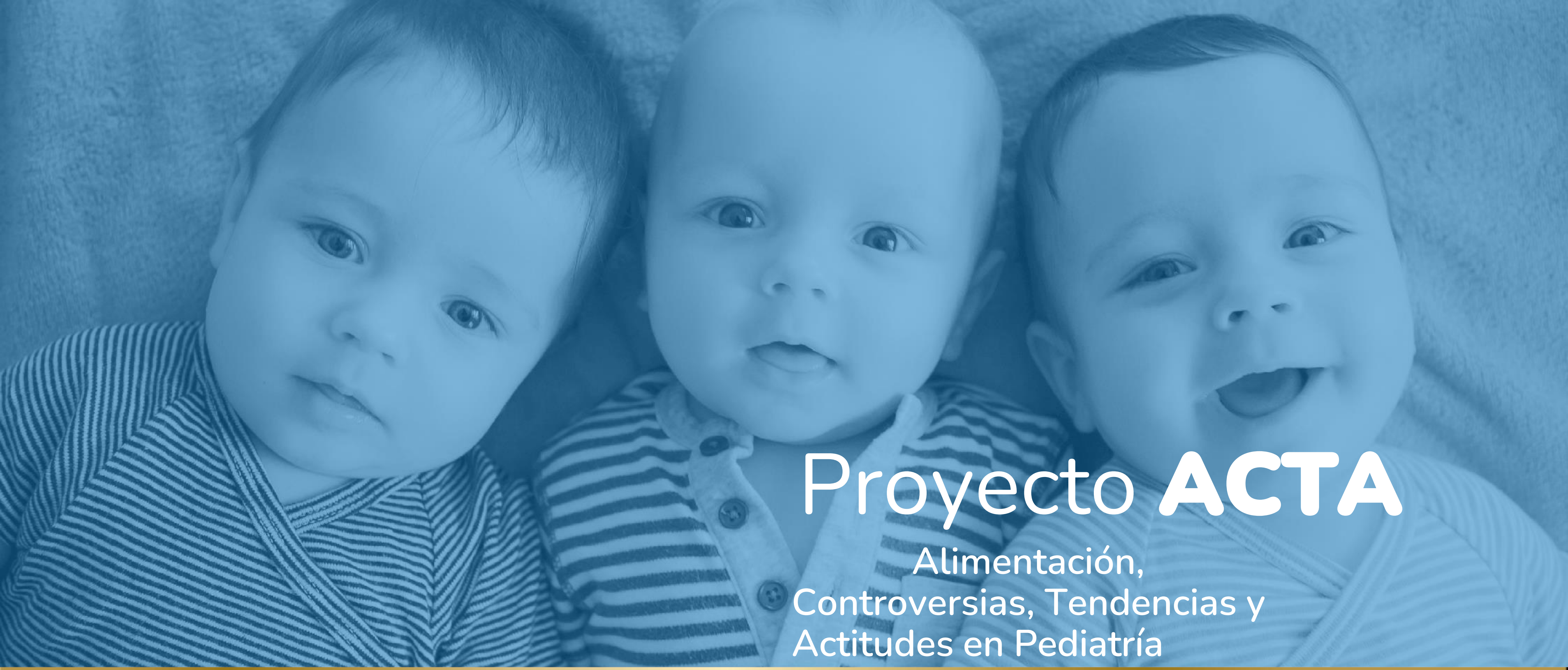

Proyecto **ACTA**

Alimentación,
Controversias, Tendencias y
Actitudes en Pediatría

Cuestionario de práctica clínica

Datos personales

1. Género ☐ Femenino ☐ Masculino

2. Edad _______ **años**

3. Provincia donde ejerce

4. Tipo de trabajo

☐ Público ☐ Privado ☐ Ambos

5. Centro de trabajo

☐ Medio rural (municipio de < 2.000 habitantes)

☐ Medio semiurbano (municipio entre 2.000 y 10.000 habitantes)

☐ Medio urbano: (municipio con más de 10.000 habitantes)

6. Tipo de centro en el que trabaja (marque todos los necesarios)

☐ Centro de salud Hospital

☐ Consulta privada

☐ Otros. Especificar ____________________

Práctica clínica

7. ¿Cuántos pacientes ≤2 años suele visitar en un mes típico?

|_|_|_| pacientes ≤2 años / mes

A continuación, se presentan de forma breve, 10 temas relacionados con la alimentación de los niños menores de 2 años que tienen cierta controversia y se realizan algunas preguntas para conocer su opinión sobre cada una de ellas. Es importante que nos de su sincera respuesta con el objetivo recoger la opinión de los pediatras españoles en relación con estos 10 temas.

Utilice el siguiente baremo:

Totalmente en desacuerdo	En desacuerdo	Ni de acuerdo ni en desacuerdo	De acuerdo	Totalmente de acuerdo
**1**	**2**	**3**	**4**	**5**

**1. Lactancia materna exclusiva y prevención de la enfermedad alérgica**	1	2	3	4	5
Es un tema sobre el que los padres me preguntan frecuentemente					
En todos los niños, la lactancia materna reduce el riesgo de alergia, especialmente si durante los primeros 6 meses se da de forma exclusiva					
En niños con un riesgo elevado de alergia por antecedentes/causas genéticas, la lactancia materna reduce el riesgo de alergia, especialmente si durante los primeros 6 meses se da de forma exclusiva					
Es un tema del que no tengo suficientes conocimientos					
La lactancia materna exclusiva no confiere protección para la prevención de la enfermedad alérgica					
Conviene evitar la exposición a cantidades pequeñas de leche de vaca (leches infantiles) durante los primeros días de vida del lactante ya que parecen incrementar la alergia a la proteína de leche de vaca					
Recomiendo a mis pacientes que alarguen al máximo la lactancia materna exclusiva para proteger a sus bebés del riesgo de alergias					
Es un tema sobre el que me gustaría tener más información					

2. Necesidad o no de hervir agua para preparar el biberón	1	2	3	4	5
Es un tema sobre la que los padres me preguntan frecuentemente					
Si el agua de consumo público sólo se somete a la cloración o la filtración rápida, como ocurre en muchos municipios, debe someterse a ebullición para inactivar los quistes u ooquistes que puedan contener					
La duración de la ebullición debe ser de sólo 1 minuto para evitar el riesgo añadido del excesivo aporte iónico					
Es un tema del que no tengo suficientes conocimientos					
Me preocupa que los padres preparen el biberón de sus hijos con agua de consumo público sin hervir previamente					
Recomiendo a mis pacientes que, si preparan el biberón con agua de consumo público, siempre la hiervan antes					
Considero que en nuestro país y en la actualidad, esta medida (hervir el agua de consumo público antes de preparar el biberón) está desfasada					
Es un tema sobre el que me gustaría tener más información					

3. BLW (Baby Led Weaning) vs. alimentación complementaria tradicional	1	2	3	4	5
Es un tema sobre la que los padres me preguntan frecuentemente					
Recomiendo activamente la práctica del BLW en mi consulta					
Aconsejo a los padres que introduzcan la alimentación complementaria triturada de forma tradicional					
Es un tema del que no tengo suficientes conocimientos					
Me preocupa que el lactante que se alimenta con BLW no reciba suficiente variedad de nutrientes y en una cuantía suficiente					
Advierto a los padres del peligro de atragantamiento del BLW					
El BLW fomenta y promueve el desarrollo psicomotor del lactante permitiéndole desarrollar las distintas habilidades que necesitará para comer					
El BLW mejora la transición a la alimentación sólida al ofrecer texturas variadas y favorecer aptitudes masticatorias					
El BLW previene la obesidad al ser el niño el que gestiona la cantidad de alimento que toma y las sensaciones de saciedad					
La mayoría de mis familias hacen BLW para introducir la alimentación complementaria					
Es un tema sobre el que me gustaría tener más información					

2. Introducción precoz de nutrientes potencialmente alergénicos y prevención de alergia	1	2	3	4	5
Es un tema sobre la que los padres me preguntan frecuentemente					
No existe evidencia en la actualidad que justifique el retraso en la introducción de alimentos potencialmente alérgicos en la introducción de la alimentación complementaria en niños sanos					
Solo tiene sentido retrasar la introducción de alimentos potencialmente alérgicos en la introducción de la alimentación complementaria en niños con antecedentes de asma, atopia o alergia alimentaria					
Es un tema del que no tengo suficientes conocimientos					
Prefiero esperar a que mis pacientes tengan 1 año para introducirles alimentos como el huevo o los frutos secos triturados para evitar problemas de alergia					
En niños con antecedentes familiares de celiaquía recomiendo retrasar la introducción del gluten en su dieta más allá de los 6 meses					
Es un tema sobre el que me gustaría tener más información					

5. Tipo de leche durante los 2 primeros años de vida (leches vegetales, de cabra, de vaca...)	1	2	3	4	5
Es un tema sobre la que los padres me preguntan frecuentemente					
La leche de vaca no debería introducirse como principal aporte lácteo antes de los 12 meses, aunque sí se puede añadir antes en cantidades pequeñas					
El empleo de leches vegetales no infantiles de forma exclusiva en el niño de corta edad (1-3 años) entraña riesgos graves para la salud					
Es un tema del que no tengo suficientes conocimientos					
Considero que no existen diferencias significativas en el crecimiento y desarrollo de niños alimentados con leche de fórmula elaborada a base de leche de cabra o vaca, por lo que sus cualidades nutricionales pueden considerarse similares					
Prefiero que los niños pequeños (>1 año) consuman leche vegetal (avena, almendras, arroz, coco...) en lugar de leche de vaca					
La cantidad razonable recomendada en una dieta diversificada y completa del niño de 1 a 3 años debería ser de dos raciones de lácteos (equivalentes a 2 vasos de 200 ml de leche de vaca)					
Recomiendo cambiar a la leche desnatada en aquellos niños entre 12 meses y 2 años con sobrepeso o si tienen familiares con colesterol elevado u otros factores de riesgo relacionados para la salud					
Es un tema sobre el que me gustaría tener más información					

6. Consumo de proteínas en los dos primeros años de vida	1	2	3	4	5
Es un tema sobre la que los padres me preguntan frecuentemente					
Creo que actualmente los niños de 6 meses a 2 años toman demasiadas proteínas en su dieta diaria					
En niños de 6 meses a 2 años, es preferible el consumo de proteína de origen animal que vegetal					
No es necesario que todos los días el menú de los niños contenga carne, ya que puede alternarse con los otros grupos de alimentos proteicos					
A las madres y padres les preocupa que los niños no tomen suficientes proteínas					
Es un tema del que no tengo suficientes conocimientos					
Generalmente aconsejo que los niños tomen sobre todo carnes blancas, por su menor contenido en grasas					
Un exceso de ingesta de proteínas en edades tempranas aumenta el riesgo de padecer obesidad en el futuro					
Recomiendo un aumento de la ingesta de proteínas en aquellos niños en edad escolar que tienen una elevada actividad física					
Es un tema sobre el que me gustaría tener más información					

7. La sal en la dieta durante los 2 primeros años de vida	1	2	3	4	5
Es un tema sobre la que los padres me preguntan frecuentemente					
Antes del año, no es recomendable añadir sal a los alimentos durante el periodo de alimentación complementaria					
Después del año es recomendable cocinar con poca sal, preferiblemente sal yodada					
Es un tema del que no tengo suficientes conocimientos					
Generalmente sugiero a mis familias que añadan sal a las comidas de los niños para que estén más sabrosas y coman mejor, independientemente de la edad					
Una ingesta excesiva de sodio en la infancia temprana puede programar el desarrollo de una presión arterial más elevada en las etapas posteriores de la vida					
Es un tema sobre el que me gustaría tener más información					

8. El azúcar en la dieta durante los 2 primeros años de vida	1	2	3	4	5
Es un tema sobre la que los padres me preguntan frecuentemente					
El consumo de azúcar en niños es una de las preocupaciones más comunes de los padres					
Hasta que los niños no cumplen 2 años deben consumir ningún tipo de azúcar añadido					
El consumo habitual y continuado de azúcar en la infancia temprana puede ser causa de diabetes en el futuro					
Es un tema del que no tengo suficientes conocimientos					
El consumo habitual y continuado de azúcar en la infancia temprana puede alterar la percepción de los sabores de las comidas en los niños					
Creo que gran parte de los problemas de obesidad en la infancia se podrían evitar moderando el consumo de azúcar en edades tempranas					
Recomiendo una mayor ingesta de azúcar en niños en edad escolar que tienen una gran actividad física					
Es un tema sobre el que me gustaría tener más información					

9. Dieta vegetariana durante los primeros 2 años de vida	1	2	3	4	5
Es un tema sobre la que los padres me preguntan frecuentemente					
En niños entre 6 meses y 2 años, las dietas vegetarianas planificadas de manera apropiada son saludables, nutricionalmente adecuadas, y pueden proporcionar beneficios para la salud en la prevención y en el tratamiento de ciertas enfermedades					
Considero que es arriesgado, desde un punto de vista nutricional, someter a los niños entre 6 meses y 2 años a dietas vegetarianas por el elevado riesgo de anemia o déficit de vitamina. B12 que pueden tener					
Una dieta sin alimentos de origen animal no puede cubrir las necesidades nutricionales y energéticas de los niños de 6 meses a 2 años					
Es un tema del que no tengo suficientes conocimientos					
Mis pacientes vegetarianos tienen un mejor estado general de salud que los pacientes con una dieta omnívora					
Recomiendo suplementar la dieta de mis pacientes vegetarianos (6 meses-2 años) con vitamina B12					
Recomiendo suplementar la dieta de mis pacientes vegetarianos (6 meses-2 años) con hierro					
Recomiendo suplementar la dieta de mis pacientes vegetarianos (6 meses-2 años) con vitamina D					
Es un tema sobre el que me gustaría tener más información					

10. Alimentos ecológicos en los primeros 2 años de vida	1	2	3	4	5
Es un tema sobre la que los padres me preguntan frecuentemente					
Prefiero que mis pacientes de 6 meses a 2 años consuman alimentos ecológicos					
Considero que los alimentos ecológicos son mejores porque tienen un mejor perfil nutricional					
Creo que los alimentos ecológicos son una moda y que su consumo no tiene impacto en la salud de mis pacientes					
Es un tema del que no tengo suficientes conocimientos					
Las propiedades nutricionales de los alimentos ecológicos son las mismas que las de los alimentos no ecológicos					
Los padres que compran alimentos ecológicos a sus hijos lo hacen porque consideran que les hace ser, en cierto modo, mejores padres					
A las familias les digo que no hace falta que compren alimentos ecológicos para sus hijos					
Es un tema sobre el que me gustaría tener más información					
