# Supplementary material for: Clinically Relevant Topics and New Tendencies in Childhood Nutrition during the First 2 Years of Life: A Survey among Primary Care Spanish Paediatricians
Source: Nutrients. 2024 Jul 5;16(13):2146. doi: 10.3390/nu16132146 (PMC11243411; doi:10.3390/nu16132146)
Supplement: Supplementary file 1 [file nutrients-16-02146-s001.zip › Supplementary material Table S2 ACTA study.pdf]

Supplementary material

Table S2. General characteristics of participants

Data	Number (%)
Gender	
Males	133 (54.3)
Females	109 (44.5)
Missing	3 (1.2)
Age distribution, years	
<30	1 (0.4)
31-40	21 (8.6)
41-50	43 (17.5)
51-60	83 (33.9)
61-70	90 (36.7)
>70	3 (1.2)
Missing	4 (1.6)
Type of work	
Public	81 (33.1)
Private	90 (36.7)
Both	63 (25.7)
Missing	11 (4.5)
Working center location	
Urban area	226 (92.2)
Semi-urban area	17 (6.9)
Rural area	1 (0.4)
Type of working center	
Outpatient hospital consultations	130 (53.1)
Private clinic	113 (46.1)
Other	24 (8.8)
